# Timing control of gait: a study of essential tremor patients vs. age-matched controls

**DOI:** 10.1186/s40673-016-0043-5

**Published:** 2016-03-02

**Authors:** Ashwini K. Rao, Elan D. Louis

**Affiliations:** Department of Rehabilitation & Regenerative Medicine (Physical Therapy), College of Physicians and Surgeons, Columbia University, New York, NY USA; G.H. Sergievsky Center, College of Physicians and Surgeons, Columbia University, New York, NY USA; Department of Neurology, Yale School of Medicine, Yale University, New Haven, CT USA; Department of Chronic Disease Epidemiology, Yale School of Medicine, Yale University, New Haven, CT USA; Center for Neuroepidemiology and Clinical Neurological Research, Yale School of Medicine, Yale University, New Haven, CT USA; Neurological Institute, 8th Floor, 710 West 168th Street, New York, NY 10032 USA

**Keywords:** Essential tremor, Gait, Timing control, Cerebellum

## Abstract

**Background:**

Essential tremor (ET) is a common movement disorder characterized by kinetic, postural and intention tremors. Mounting evidence suggests an underlying dysfunction of the cerebellum or cerebellar system. While few recent studies report impairments in timing control of finger movements in ET, timing control of gait has not been examined to date. We compared timing control of gait in ET patients vs. controls, and further assessed the association of these timing impairments with tremor severity among the ET patients. One-hundred-fifty-five ET patients and 60 age-matched controls underwent a comprehensive neurological assessment and gait analysis, which included walking at a criterion step frequency (cadence) with a metronome (timing production) and walking at a criterion step frequency after the metronome was turned off (timing reproduction). Outcomes of interest for both conditions were timing accuracy (measured by cadence error) and timing precision (measured by cadence variability). We also assessed cadence and step time across conditions.

**Results:**

Cadence was lower in ET patients than controls (*p* < 0.03), whereas step time was similar for ET patients and controls. Accuracy (cadence error) and precision (cadence variability) were not different in ET patients compared with controls. Cranial tremor score was significantly associated with cadence (timing production condition, *p* = 0.003 and timing reproduction condition, *p* = 0.0001) and cadence error (timing production condition, *p* = 0.01). Kinetic tremor and intention tremor scores were not associated with gait measures.

**Conclusions:**

ET patients do not demonstrate impairments in timing control of gait as compared with matched controls. Prior work shows that patients with cerebellar dysfunction demonstrate selective impairments in timing of discrete movements (such as finger tapping) but not continuous movements (such as circle drawing). Taken together, these results support the hypothesis that the cerebellum may be important for timing control of discrete rather than continuous movements.

## Background

Essential tremor (ET) is a highly prevalent neurological disorder characterized by a variety of tremors [1]. The diagnosis of ET is based on history and neurological examination. Kinetic tremor (i.e., tremor occurring during voluntary movement), particularly of the upper limbs, is the most recognizable feature of ET. Kinetic tremor in ET may have an intentional component, which is most pronounced just before movement termination [2]. Postural tremor may also be seen, especially when the arms are maintained against gravity [1, 3]. Tremor in ET is associated with difficulties in the performance of activities of daily living [4, 5].

In addition to tremor, ET also presents with characteristic balance and gait abnormalities [6–8]. Gait abnormalities include decreased speed (beyond that seen with aging) and impaired dynamic balance – ET patients spend greater time in double support while walking at a preferred speed and have more mis-steps during tandem gait [7, 9]. Gait impairments worsen during the performance of a secondary cognitive task during gait, particularly in ET patients with cognitive limitations [10]. Gait impairments in ET result in an increase in fall risk [11, 12].

Clinical features of ET such as intention tremor and tandem gait abnormalities may be due to cerebellar dysfunction [7, 9, 10]. Imaging studies have noted several abnormalities in the ET cerebellum [13–15], and recent neuropathological studies have catalogued a host of changes in the ET cerebellar cortex [16, 17].

More broadly, cerebellar dysfunction has been suggested to result in impaired control of movement timing [18], particularly for rapid finger and hand movements [19, 20]. In a comprehensive review of the role of the cerebellum in motor control, Manto and colleagues suggest that the cerebellum is particularly important for controlling timing of movements that are marked by discrete events (e.g. finger tapping) [18]. In two studies of repetitive finger tapping movements, ET patients demonstrated reduced speed [21], and reduced accuracy [22]. In addition, ET patients with head tremor were impaired at a predictive task in which subjects were required to perform a discrete arm movement to intercept a moving target [23]. However, timing control of gait has not been examined in ET. The purpose of this study was (1) to examine if gait timing control was impaired in ET patients compared with age-matched controls while walking at a self-selected preferred speed and while walking with an external timing cue (metronome) and (2) to examine whether timing impairments are related to clinical measures of disease severity (i.e., kinetic tremor score, intention tremor score, cranial tremor score).

## Results

### Demographic and clinical characteristics

There were 225 participants. Of these, 10 participants were excluded because their mMMSE score was < 40. The final sample of 215 participants included 155 participants with ET and 60 spousal controls. Demographic and clinical data are presented in Table 1. Age, sex distribution, height, weight and leg length were similar across groups (*p* ≥ 0.21 for all variables). For ET participants, mean age of tremor onset was 41.1 (±22.8) years. Examination of cranial tremor revealed that 96 out of 155 ET patients (61.9 %) had cranial tremor in one or more locations. Forty-three out of 155 ET patients had neck tremor (27.7 %). Thirty-eight (24.5 %) ET patients had postural instability, as indicated by a score of two or higher on the UPDRS retropulsion test. With regard to lower limb kinetic tremor, 9 ET patients (6 %) had moderate amplitude or higher tremor in both legs (score ≥ 2 out of 4).

**Table 1:** Demographic and clinical characteristics of participants

Outcome	ET	Control	Significance
n	155	60	
Age, years (SD)	81.9 (8.5)	80.1 (6.4)	0.21^a^
Sex, Female (%)	92 (59.4 %)	40 (66.7 %)	0.40^b^
Height, meters (SD)	1.7 (0.4)	1.7 (0.3)	0.51^c^
Weight, Kg (SD)	68.4 (17.9)	68.1 (13.7)	0.91^c^
Leg Length, meters (SD)	0.93 (0.06)	0.93 (0.06)	0.45^c^
Age at tremor onset, years (SD)	41.1 (22.8)	NA	NA
Cranial Tremor Score, N (%)*			
0	57 (36.7 %)	NT	NA
1	43 (27.7 %)		
2	42 (27.1 %)		
3	11 (7.1 %)		
Kinetic Tremor Score, Mean ± SD Range	13.74 ± 4.24 1–24	NT	NA
Intention Tremor Score, Mean ± SD Range	1.39 ± 0.64 0–2.5	NT	NA
Neck Tremor, N (%)			
0 Absent	112 (72.2 %)	NT	NA
1 Present	43 (27.7 %)		
Postural Stability Retropulsion Test, N (%)			
0 Normal	4	NT	NA
1 Recovers unaided	113		
2 Would fall if not caught	30		
3 Falls spontaneously	8		
4 Unable to stand	0		

*Data unavailable on two ET cases

*NA* not applicable, *NT* not tested, *SD* standard deviation

a = independent t-test; b = Chi-square test; c = Mann–Whitney U test

### Quantitative gait measures

While walking at a self-selected preferred speed, mean step time was similar for ET patients compared with controls (ET = 0.66 ± 0.21; Controls = 0.63 ± 0.11, t = 0.81, p = 0.42). Step time coefficient of variation was also similar across groups (ET = 7.21 ± 14.01; Controls = 6.39 ± 9.18, t = 0.39, p = 0.69). These results suggest that ET patients did not demonstrate a deficit in gait timing while walking at a preferred speed.

Mean (standard deviation) for gait measures during the externally cued (metronome) conditions are presented in Table 2. Mean step time was similar for ET patients and controls across conditions (F = 0.03, *p* = 0.95). Similarly, step time coefficient of variation was not different across groups and conditions (F = 0.06, *p* = 0.81). Cadence (step frequency) was lower in ET patients compared with controls under both conditions (timing production and reproduction). This was confirmed statistically by a main effect of group (F = 4.46, *p* = 0.03). Cadence error (index of accuracy) was similar in patients with ET and controls (main effect of group, F = 0.001, *p* = 0.99). Similarly, cadence standard deviation (an index of precision) did not reveal differences across groups (main effect of group, F = 0.11, *p* = 0.75). These data suggest that gait timing was not impaired in ET patients while walking with an external auditory cue (i.e., a metronome).

**Table 2:** Mean and standard deviation (SD) for gait measures across conditions and groups

Gait measure	Step frequency production		Step frequency reproduction	
	ET	Control	ET	Control
	Mean (SD)	Mean (SD)	Mean (SD)	Mean (SD)
Step Time	0.68 (0.19)	0.62 (0.18)	0.66 (0.16)	0.69 (0.17)
Step Time Coefficient of Variation	9.36 (16.53)	6.01 (5.27)	6.21 (21.82)	7.97 (9.97)
Cadence	92.21 (25.02)*	100.58 (16.64)	96.69 (19.31)*	102.14 (17.71)
Cadence Error	−0.04 (0.27)	−0.01 (0.09)	−1.27 (12.42)	−1.24 (9.17)
Cadence Standard Deviation	5.76 (26.77)	7.31 (19.98)	2.46 (2.39)	2.14 (2.16)

Statistically significant effects are highlighted with an asterisk

### Association of gait and clinical measures

Results of the linear regression analyses are shown in Table 3. Cranial tremor score was a significant independent predictor of cadence in the production and reproduction conditions: cadence decreased as severity of cranial tremor increased. Cranial tremor score was also a significant predictor of cadence error in the production, but not during the reproduction condition: cadence error increased as severity of cranial tremor increased. The magnitude of the associations between cranial tremor and gait measures ranged from small (0.18) to moderate (0.31). Similarly, postural stability (measured by the retropulsion test) was a significant predictor of cadence in both conditions, and a significant predictor of cadence error in the production condition. In contrast, kinetic tremor score and intention tremor score were not significantly associated with any gait measures (Table 3). Presence of neck tremor was also not associated with gait (*p* > 0.26). Age was not a significant predictor in any of the regression models (*p* > 0.3 in all models).

**Table 3:** Results of the linear regression analysis of tremor, postural instability and gait measures

	Cranial tremor score	Kinetic tremor score	Intention tremor score	Postural instability
PRODUCTION Cadence				
Standardized _β_	−0.26	−0.14	−0.05	−0.48
t-statistic (significance)	−2.99 (**0.003**)	−1.63 (0.11)	−0.59 (0.55)	6.07 (**0.0001**)
PRODUCTION Cadence Error				
Standardized _β_	0.31	0.13	0.13	−0.31
t-statistic (significance)	3.66 (**0.0001**)	1.41 (0.16)	1.49 (0.14)	3.7 (**0.0001**)
REPRODUCTION Cadence				
Standardized _β_	−0.18	−0.06	−0.11	−0.35
t-statistic (significance)	−2.25 (**0.01**)	−0.71 (0.48)	−1.29 (0.19)	4.52 (**0.0001**)
REPRODUCTION Cadence Error				
Standardized _β_	0.09	0.03	0.07	−0.11
t-statistic (significance)	1.16 (0.25)	0.41 (0.68)	0.85 (0.39)	0.13 (0.89)

Significant differences appear in bold font

## Discussion

Gait and balance impairments in ET have been described in numerous studies. These problems are not simply sub-clinical phenomena, observed only in the laboratory [7, 10, 12]. In some patients, the problems have the potential to increase falls and fear of falls [11, 12]. The presence of specific gait impairments (including decreased speed and impaired dynamic balance) suggests that these impairments may have a cerebellar origin [7, 8, 24–27].

Cerebellar dysfunction is suggested to result in impairments in the timing control of movements [18]. In ET, performance of motor timing tasks, such as repetitive finger tapping, is impaired [21, 22, 28]. In particular, finger-tapping movements are slower [21, 22] and more variable compared with controls [22]. However, these motor impairments (slow and variable movements) were not correlated with clinical markers of tremor severity [21, 22]. Target interception using a key press movement was also impaired in ET patients with head tremor, suggesting that the cerebellum may be implicated in predictive timing of discrete movements [23].

In the present study, we examined whether motor timing was impaired during gait in a large sample of 215 subjects, which included 155 ET subjects. The results of our study show that timing control during gait is not impaired in ET in comparison with matched controls. Cadence (step frequency) was lower in ET patients compared with controls while producing and reproducing criterion cadences (step frequencies). Lower cadence in patients with ET may be a function of slower gait speed, which has been previously reported [7, 29]. However, cadence error was similar across the two groups, indicating that both groups were comparably accurate at producing and reproducing criterion cadence during gait. Similarly, cadence standard deviation (a measure of precision) was also similar in ET patients and controls. Analysis of step time and step time variability yielded similar results: patients with ET performed similar to controls. Our results, in the context of previous studies on motor timing deficits in ET, suggest that timing control impairment in ET may be specific to some activities but not others.

Why is timing control in ET impaired during finger tapping but not during gait? One explanation is that discrete motor tasks such as finger tapping may have distinct control mechanisms in comparison with continuous motor tasks such as circle drawing and gait [30–32]. Support for this hypothesis comes from results showing that timing variability in discrete movements, such as finger tapping, is not correlated with timing variability during circle drawing movements [30–32] or with variability during gait [33]. Additional support for this hypothesis comes from imaging studies [34, 35]. Functional imaging of healthy subjects while performing discrete and continuous movements indicates that the cerebellar vermis [35], the dentate nucleus and cerebellar homunculus [34] are selectively active during discrete rather than continuous movements. Control mechanisms for discrete movements may include an explicit timing representation. In contrast, timing in continuous movements is thought to be an emergent property of movement control [32].

Patients with cerebellar lesions have deficits in timing of discrete (discontinuous) movements (such as finger tapping) but not continuous movements (such as circle drawing and gait) [31, 36]. These results suggest that the cerebellum may be involved in timing control of discrete movements that require an explicit timing representation. In contrast, timing deficits in continuous movements are pronounced in patients with basal ganglia disease such as Parkinson’s disease (PD) and Huntington’s disease (HD) [37–40].

The results of our regression analysis suggest that cranial tremor (tremor in midline structures) was associated with gait measures (cadence and cadence error). Postural instability (measured by the retropulsion test) was also associated with gait measures. In contrast, kinetic tremor and intention tremor (tremor in the extremities) were not associated with gait measures. These results are similar to our prior work and suggest that gait impairments may be related to pathology in midline cerebellar structures which may be implicated in head/neck control and postural stability [9]. In our results, presence of neck tremor was not associated with gait. The results of this study, along with our prior work [7, 9–12, 41] indicate that gait impairments in ET primarily include deficits in speed and balance control, whereas timing control during gait is spared. Future work should compare timing control in discontinuous tasks (such as finger tapping) with continuous tasks (such as gait or circle drawing) in order to further understand the role of the cerebellum in timing control of movements. In addition, imaging of the cerebellum during observation or imagery of gait may be important in further elucidating the role of the cerebellum during gait. Recent imaging studies of motor imagery or action observation of gait have shown that similar cortical and sub-cortical regions are active during performance of gait and observation or imagery of gait [42, 43]. However, results of imaging of observation or imagery of gait must be interpreted with caution because imaging or observation of gait do not involve balance and postural control, which is an inherent aspect in the control of functional gait.

One potential limitation is that our study tested production and reproduction of two criterion cadences. Future work should test production and reproduction at additional criterion cadences in order to generalize our findings. Second, we did not administer a test of timing control in a discrete task such as finger tapping. Given the extensive battery of clinical assessments (~ 3 h), we limited the quantitative motor assessment in order to minimize subject fatigue. The strengths of our study include a large sample size and uniform administration of assessments across participants.

## Conclusions

We examined timing control during gait in patients with ET as compared with control subjects. Our results indicate that patients with ET do not have impairment in gait timing control compared with control subjects. Our results are in contrast with studies on repetitive finger tapping movements, in which patients with ET demonstrate less accuracy and precision. One explanation for the contrasting results is that control mechanisms for finger tapping (a discrete task) and gait (a continuous task) may be distinct, as proposed in the literature [31, 32]. The cerebellum may be implicated in the timing control of discrete motor tasks (such as finger tapping) rather than continuous tasks (such as circle drawing and gait).

## Methods

### Subjects

Participants were enrolled in the study, the Essential Tremor Centralized Brain Repository (ETCBR), as future brain donors. Participants were recruited through (a) advertisements in the International Essential Tremor Foundation website and newsletter; (b) advertisement on the Tremor Action Network website; and (c) the ETCBR website (www.essentialtremor.us). We recruited patients with ET and spousal controls living across the United States. The diagnosis of ET was confirmed (by EDL) using published criteria (moderate or greater amplitude tremor during three or more activities, or a head tremor in the absence of PD or another known cause. Spousal controls were recruited if they did not have a diagnosis of ET or another movement disorder. Participants with dementia (defined as a score <40 on the Modified Mini-Mental Status Examination (mMMSE)] [10], other neurological disorders (including stroke, PD), or orthopedic disorders that impair walking (such as hip or knee replacement), were excluded from the study. We excluded participants with dementia, as they would not have been able to follow task instructions. All participants (*N* = 215; 155 patients with ET, 60 spousal controls) signed a written informed consent, approved by Columbia University institutional ethics committee.

### Assessment

Testing was completed at each participant’s home and took approximately 3 h. This testing included a clinical assessment and quantitative gait assessment. Testing at home allowed us to recruit participants who would not have been able to travel to a medical center for testing, and allowed us to examine participants in a familiar environment. To minimize testing variability, we ensured that all participants had access to a well-lit hallway long enough to accommodate the GAITrite® mat. Participants were given rest periods during the assessment, as needed, to minimize fatigue.

### Clinical assessment

The clinical assessment included the collection of demographic and clinical data (including age, sex, and age at tremor onset). We administered the mMMSE [44]. This expanded version of the Folstein Mini-Mental State Examination has demonstrated validity and reliability (range = 0–57, higher scores indicate better performance) [45].

The clinical assessment included a detailed videotaped examination. The kinetic tremor score was computed as the sum of the 0–3 ratings for five videotaped items (pouring, drinking, using a spoon, writing, finger to nose test) that were performed with each arm (range 0–30, higher scores indicating greater tremor severity). Intention tremor of the arms was scored during the finger to nose test (0 = no trouble, 1 = simple swerve of movement, 2 = moderate tremor with amplitude < 10 cm, 3 = tremor with amplitude between 10 cm and 40 cm, 4 = severe tremor with amplitude > 40 cm), and the intention tremor score ranged from 0–8. Jaw, voice and neck tremors were assessed on videotape examination with participants seated in front of a camera, and were scored as present or absent. Jaw tremor was tested with the mouth closed, mouth slightly open, and during sustained phonation and speech. Voice tremor was tested during sustained phonation, reading a paragraph, and during speech. Neck tremor was distinguished from dystonia by the absence of tilting and sustained neck deviation, or hypertrophy of neck muscles. We computed a cranial tremor score for each ET patient as the number of locations in which such tremor was present (jaw, voice, neck, range = 0–3). Postural stability was assessed with retropulsion test of the Unified Parkinson’s Disease Rating Scale (UPDRS). The test was scored as 0 = normal, 1 = recovers unaided, 2 = would fall if not caught, or 3 = falls spontaneously. Kinetic tremor was assessed during the toe-to-pen test with each foot, and scored as follows: 0 = no visible tremor, 0.5 = very low amplitude tremor and almost never present, 1 = low amplitude or intermittent tremor, 1.5 = moderate amplitude oscillatory tremor, but only sometimes, 2 = moderate amplitude oscillatory tremor usually present, 3 = large amplitude, 4 = extremely large tremor. We computed a kinetic tremor score in both legs by combining the tremor score in each leg and dividing by 2 in order to maintain the scoring system. We also recorded subject weight (in Kilograms), height (in meters) and measured leg length (distance, in meters, from the greater trochanter to the floor).

### Quantitative gait assessment

We used the GAITrite computerized mat to assess gait. The mat (4.6 m length) was placed in the middle of a quiet 10 m long hallway. Participants began walking 2 m before the beginning of the mat and stopped 2 m beyond the end of the mat. This allowed us to record steady-state gait without the influence of initiation and termination. The GAITrite mat has pressure sensors that capture the location and timing of individual footfalls, and the accompanying software allows for computation of spatio-temporal outcome measures. Performance was tested under three conditions, with five trials per condition. The conditions were: (a) standard walk, during which subjects were asked to walk at their self-selected preferred speed; (b) timing production, during which participants were asked to step in time with the beat of a metronome presented during each trial. The metronome beat was set at the cadence (i.e., step frequency per minute) recorded during standard walk; and (c) timing reproduction, during which participants were presented with a metronome beat for 10 s and asked to step in time with the beat after the metronome was turned off. We requested participants not to use assistive mobility aids (such as canes or walkers) during the testing.

Data were analyzed by AKR, who was blinded to group assignment of participants. Since we have reported data for the standard walk condition previously [7, 10], here we present data only for the two metronome conditions. Criterion cadence during the metronome conditions was computed from cadence (step frequency) during standard walk condition. We tested performance under two conditions: (1) cadence measured during standard walk, (2) 110 % of cadence measured during standard walk. The primary outcomes of interest were (1) mean step time, in seconds, defined as the time between first contact of one foot to the first contact of the other foot, (2) step time coefficient of variation, computed as standard deviation of step time divided by mean step time and expressed as a percentage, (3) mean cadence (defined as step frequency per minute), (4) mean cadence error (computed as the difference between criterion cadence and measured cadence), and (5) cadence standard deviation, computed as the within-subject standard deviation in cadence across trials in each testing condition. Participants walked an average of 10 steps per trial, allowing us to use ~50 steps for computing gait variability, which is reported to be adequate [46].

### Statistical analysis

Demographic and clinical variables across groups were analyzed by One Way Analysis of Variance (ANOVA). When variables were not normally distributed, we used non-parametric (Mann–Whitney U) statistics. Group differences while walking at a self-selected preferred speed were analyzed using independent sample t-tests. Group differences for primary outcomes were analyzed using general linear model procedures with group (control and ET) and condition (timing production and reproduction) as factors. In order to examine the association between gait measures and measures of tremor severity (kinetic tremor score, intention tremor score, cranial tremor score), we conducted independent linear regression analysis with tremor scores as predictors of gait measures. We also examined the association between postural stability and gait, using linear regression analysis. We included age in all regression models. In addition, we examined if there were differences in gait between ET patients with neck tremor and those without neck tremor. Analyses were performed in SPSS (version 22.0) by AKR and we used 0.05 as level of significance.

## Abbreviations

ET: essential tremor
ETCBR: essential tremor centralized brain repository
mMMSE: modified mini mental state examination
PD: parkinson’s disease
